# Optimizing Waterborne Polyacrylate Coating for Controlled-Release Fertilizer Using a Wurster Fluidized Bed and Its Effects on Rice Yield

**DOI:** 10.3390/polym17212816

**Published:** 2025-10-22

**Authors:** Cong Zhao, Xuefang Huang, Juanling Wang, Changwen Du

**Affiliations:** 1 Shanxi Institute of Organic Dryland Farming, Shanxi Agricultural University and Key Laboratory of Sustainable Dryland Agriculture of Shanxi Province, Taiyuan 030031, China; zhaocong502@126.com (C.Z.); 13653667079@163.com (X.H.); juanling_wang@sxau.edu.cn (J.W.); 2 Key Laboratory of Sustainable Dryland Agriculture (Co-Construction by Ministry of Agriculture and Rural Affairs and Shanxi Province), Jinzhong 030801, China; 3 National Agricultural Environment Observation and Experimental Station in Jinzhong, Taiyuan 030031, China; 4 The State Key Laboratory of Soil and Agricultural Sustainability, Institute of Soil Science Chinese Academy of Sciences, Nanjing 211135, China

**Keywords:** waterborne polyacrylate, eco-friendly coating, controlled release fertilizer, rice actual yield

## Abstract

The efficacy of coated fertilizers in enhancing nutrient use efficiency and reducing environmental impacts depends on their coating properties. This study developed three biodegradable, waterborne polyacrylate latexes (A, B, and C) as eco-friendly coatings for controlled-release fertilizers (CRFs) using the Wurster fluidized bed process. The latexes were synthesized with varying hard-to-soft monomer ratios and cross-linked with 2 wt% aziridine to investigate how monomer composition affects coating properties and nutrient release. The results showed that coating B, which had an intermediate hard-to-soft monomer ratio, demonstrated optimal properties. It exhibited the lowest swelling capacity (2.54% at 60 °C), a suitable glass transition temperature (15.34 °C), and the slowest nutrient release, with cumulative nitrogen release remaining below 60% after 11 days in water at 40 °C. In field trials, the fertilizer coated with material B produced the highest rice yield among tested domestic CRF brands. It also achieved a significant 19.1% yield increase compared to a single basal application of conventional compound fertilizer. These findings confirm that this modified latex provides an effective and environmentally friendly solvent-free coating strategy for high-performance CRFs.

## 1. Introduction

The increasing global population and the corresponding rise in food demand have necessitated the development of sustainable agricultural practices [1,2]. It is forecasted that food demand will increase by 70% of the current requirements to satisfy the need of the booming population, which is projected to hit 9.5 billion people by 2050 [3]. Fertilizers, one of the most important products in the agrochemical industry, play a critical role in sustainable agriculture and global food security, which contributes more than 60% to yield [4,5]. In response to increasing food requirements, farmers are applying enormous quantities of fertilizers to maximize the agricultural yield. However, due to the rapid release rate and short duration of conventional fertilizers, the use efficiency is currently very low. Alarmingly, nearly 70% of nitrogen (N) and 80~90% of phosphorus (P) are lost through leaching, volatilization, or runoff [6,7]. The excessive application of conventional fertilizers leads to serious environmental problems such as eutrophication, groundwater contamination, soil acidification, and greenhouse gas emissions [8,9,10,11].

One promising method for reducing nutrient losses and environment risks involves the development of controlled-release fertilizers (CRFs). CRFs are a type of fertilizer specifically designed to release nutrients in a controlled, delayed manner that matches the nutritional requirements of plants at different growth stages. Moreover, they can save time, money, and labor because only a single application is needed. Thus, they are increasingly being recognized as an efficient, economical, and safe method of fertilizer application to alleviate the above-mentioned problems [12]. CRFs have been extensively studied and applied for decades worldwide [13,14]. Such fertilizers demonstrate many advantages over the conventional forms, such as lowered losses through volatilization or leaching, reduced fertilizer usage and application cost, and maximized utilization of the active agent from plant systems, preventing seedling damage and enhancing synergistic effects between nutrients [15,16,17].

The encapsulation of mineral fertilizers by various inorganic and/or organic materials as diffusion barriers is the main way to produce CRFs and to control the NPK release rate. Over time, a variety of materials have been adopted as CRF coatings. Initially, sulfur was tried as a coating agent, as it is an attractive candidate for coating [18,19]. However, additional expense was added to the final product due to its low wettability and adhesion to the coated core, as well as the need for conditioning materials to enhance its sealing [20]. Furthermore, any sulfur residues in the soil can react with water, thus acidifying the soil. Subsequently, hydrophobic polymer materials were considered potential candidates for CRFs, such as dichloromethane [21], ethylcellulose [22], N, N-dimethyl-formamide [23], or chloroform [24]. Though these materials are not disturbed readily by microorganisms like sulfur coatings [25], their dissolution is dependent on costly volatile organic solvents during the coating process, and these are harmful to the environment and result in secondary pollution [26]. In addition, the majority of these polymers are non-biodegradable after total nutrient release, thereby causing a new soil pollution source [20]. Hence, the research focus is shifting toward waterborne coatings, which have the advantages of nontoxicity, nonflammability, low price, and good quality compared with the traditional organic coatings [27].

Besides fertilizer, water is another key factor that plays a crucial role in agricultural production. Especially in semi-arid and arid lands, many farmlands suffer from water resource shortages, and it is necessary to improve the efficiency of water use. Therefore, a combination of superabsorbent polymers (SAPs) and CRFs became the latest trend in agriculture. Polyacrylates (PAs) are among the popular synthetic polymers for biomaterial development. Additionally, they are good candidates as coatings for CRFs when adopting waterborne coatings and for reacted layer technology when using an aqueous solution in production [28,29]. In addition to their good film-forming properties and relatively low production cost [30], these polymers are also biodegradable in the soil, degrading at a rate of 0.12–0.24% every six months [31]. Furthermore, PA-coated CRF has been reported to increase corn and wheat yields in the China [32,33]. Nevertheless, the waterborne polyacrylate coating latex is not without its flaws. For example, the coated fertilizer particles were inclined to agglomerate due to surface tackiness during storage, which could permanently degrade the coating’s performance. To reduce viscosity, three types of modified waterborne polyacrylate emulsions were synthesized by varying the ratio of soft to hard monomers. Soft monomers primarily contribute to material flexibility, whereas hard monomers improve hardness. An optimally formulated ratio can alleviate or eliminate the aforementioned issue.

In this study, the structures and properties of three modified waterborne polyacrylate emulsions, tailored by varying monomer ratios to regulate nutrient release rates, were systematically investigated. Based on laboratory results, the optimal polyacrylate formulation was selected as a coating material for CRFs and produced on a pilot scale. Its performance was subsequently evaluated in field trials comparing its effects on rice yield with those of several leading commercial slow-/controlled-release fertilizer brands in China. The main objectives were to optimize a waterborne polyacrylate-based CRF coating formulation and assess its efficacy on rice yield under practical field conditions.

## 2. Materials and Methods

### 2.1. Preparation of Polyacrylic Model Films

Three kinds of modified polyacrylate latex (A, B, and C) containing more than 65% acrylic polymer were developed by Doctor Hydrophilic Chemicals Co., Ltd. (Yizheng, China) in collaboration. The selected monomers, additives, and their corresponding proportions are shown in Table S1. Figure S1 displays the general production process. For the sake of commercial confidentiality, only the order of the hard/soft monomer ratio is listed here: C > B > A. Distilled water (20 mL) and 2 wt% cross-linker (aziridine) were slowly added to the aforementioned polyacrylate latex (20 mL) at room temperature under continuous stirring for 15 min. Then, the prepared latex (4 mL) was distributed into a 9 cm^2^ leveled glass-plate mold, dried in an oven at different temperatures (60, 70, and 80 °C) for 24 h, and formed into the model membranes on a glass plate. The model films were removed from the mold carefully and stored in a 4 °C refrigerator for use.

### 2.2. Preparation of Polymer-Coated Fertilizers

Briefly, 500 g of conventional compound fertilizers (NPK, 15-12-15), purchased from Fulilong Chemical Co., Ltd. (Zhanjiang, China), were loaded into a fluidized-bed coater (LDP-3, Jiafa Mechanic Co., Ltd., Changzhou, China) assembled with a Wurster bed on a bench scale. Before coating, fertilizer granules of 3~4 mm in diameter were sieved as the core product. The bed temperature was set at 40 ± 5 °C. After preheating at this temperature for 10 min, 100 g polyacrylate latex and 2 wt% cross-linker were added and diluted with 100 g water, which was sprayed as a coating material through a nozzle at the atomizing pressure of 0.10~0.20 MPa, and the spray rate was controlled by a peristaltic pump to a speed of 1.67 mL min^−1^. Thereafter, the coated fertilizers were dried at 60 °C in an oven for 2 h to complete the cross-linking reaction. Based on the laboratory results, the ideal coating material would be used for a pilot-scale production.

### 2.3. Characterization of Model and Coated Films

Here, 1 g of accurately weighed dry model film sample (W_d_) was immersed in 100 mL distilled water at 25 °C until completely swollen. Then, the swollen sample was taken out carefully with forceps, the excess free water on the surface was wiped out with filter paper, and the wet sample (W_w_) was weighed gravimetrically to calculate the swelling capacity using the following equation [34].

Swelling ratio = (W_w_ − W_d_)/W_d_ × 100 (1)

The glass transition temperature (*T_g_*) of the model film was analyzed by differential scanning calorimetery (DSC) on a Pyris-DSC (Perkin-Elmer, Waltham, MA, USA). Samples of approximately 6 mg were encapsulated in standard aluminum DSC pans and heated from −50 °C to 100 °C with a heating rate of 20 °C·min^−1^ under 20 mL·min^−1^ of argon gas. As a rule, two successive scans were made for every sample. All calculations were performed on the second heating cycle. The *T_g_* adopted corresponds to the inflection point in the heat-capacity jump.

The films were detached from the coated fertilizers and their surface morphology was examined using an optical microscope (BH2, Olympus, Tokyo, Japan). The structural characteristics of the coating membranes were analyzed by Fourier-transform mid-infrared photoacoustic spectroscopy (FTIR-PAS, Nicolet 380) equipped with a photoacoustic accessory (MTEC model 300, Thermo Nicolet Corporation, Madison, WI, USA). Spectra were acquired over the wavenumber range of 500~4000 cm^−1^ with a resolution of 4 cm^−1^ and a mirror velocity of 0.31 cm·s^−1^, and 32 consecutive scans were accumulated for each measurement.

### 2.4. Nutrient Release from the Coated Fertilizer in Water

The dissolution test was conducted using glass bottles under static conditions (without agitation), maintained at 40 ± 1 °C in an incubator throughout the experiment. For each replicate, accurately weighed coated granules (2 g) were immersed in 100 mL of deionized water, with three replicates performed in total. Nutrient release was assessed by monitoring the conductivity of the solution, which was measured every 24 h using a digital conductivity meter (DDS-320, Kangyi Instruments Co., Ltd., Shanghai, China). At the end of the release study, the coated fertilizer was ground into powder to determine the residual nutrient content. The release profile was normalized and expressed as the cumulative release percentage plotted against time [35].

### 2.5. Rice Yield Performance Assessment

The field experiment was conducted over June to November 2019 and 2020 at the Yingtan State Key Agro-Ecological Field Experiment Station (28°15′ N, 116°55′ E) of the Chinese Academy of Sciences in Yujiang Country, Jiangxi Province, China. The climate is subtropical monsoon with an average temperature of 18.5 °C and annual total rainfall of 1854 mm. Rainfall and temperature during the experimental period are shown in Table S2. The soil is classified as typical Haplaquept (18.2% clay, 31.3% silt, 50.5% sand), and the 0~20 cm layer has a bulk density of 1.13 g cm^−3^, a pH of 4.74, an organic C content of 16.9 g kg^−1^, and a total N content of 1.62 g kg^−1^. The late rice variety (Nongxiang 98) was transplanted at a spacing of approximately 13.2 × 29.7 cm. Each treatment included three replicates in a completely randomized block design with an individual plot of 30 m^2^. All fertilizer treatments received the same amounts of nitrogen, phosphorus, and potassium, with single basal applications of 195 kg N, 124.8 kg P_2_O_5_, and 189.3 kg K_2_O per ha. The unequal contents of phosphorus and potassium in the tested fertilizers were supplemented with calcium magnesium phosphate and potassium chloride. The types and codes of the fertilizers selected for the experiment are presented in Table 1.

Rice within 1 m^2^ was harvested at physiological maturity. The corresponding yield components of each treatment were measured, including the number of panicles per ha, grains per panicle, seed-setting rate, 1000-grain weight, and actual yield.

### 2.6. Statistical Analysis

Statistical analyses were carried out using a one-way analysis of variance (ANOVA) procedure in SPSS software (IBM SPSS Statistics version 22) to check the normal distribution and homoscedasticity and to detect differences in tested parameters. Significant differences (*p* < 0.05) between data were determined with a *t*-test and Duncan’s multiple comparisons according to the treatments.

## 3. Results

### 3.1. Traits of Model Films

**Figure 1:**
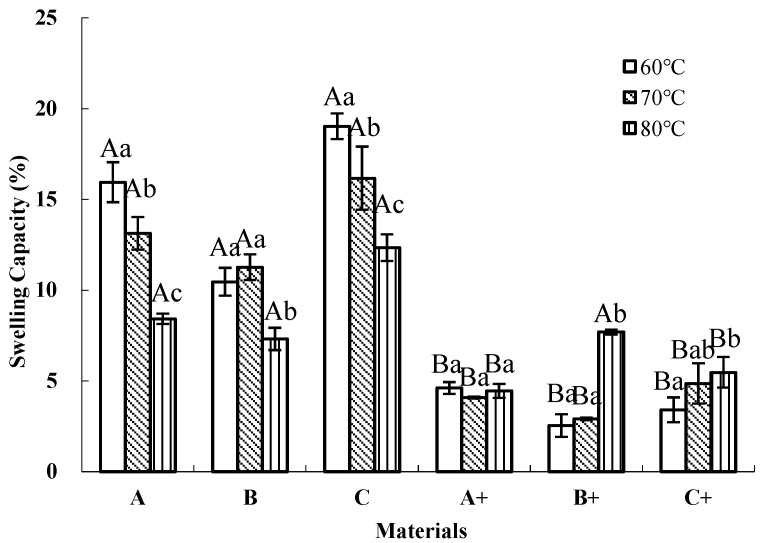
The swelling capacity of six model membranes forming under different temperatures for 24 h. Means with the same letter (capital letters for cross-linker treatment and small letters for temperature treatment) are not significantly different at *p* < 0.05 level by SPSS 22.0. Error bars indicate the standard error of the means (*n* = 3). + denotes the addition of 2 wt% cross-linker.

Figure 1 displays the effects of the cross-linker and film-forming temperature on the swelling capacity of coating materials made from waterborne polyacrylate latexes A, B, and C with 0 wt% and 2 wt% cross-linker. After the addition of the cross-linker, the swelling capacity of coating materials A and C decreased significantly (*p* < 0.05) under all forming temperatures, while that of coating material B decreased significantly (*p* < 0.05) except for when forming at 80 °C. Among them, coating material C showed the most significant (*p* < 0.05) reduction in swelling degree. Prior to the addition of the cross-linker, coating material C exhibited the highest swelling capacity among the samples at the corresponding film-forming temperature, with values ranging from 12.34% to 19.03%. However, after the cross-linker was introduced, this behavior changed significantly. The swelling capacity of material C decreased markedly and stabilized within a narrower range of 3.41% to 5.47%, no longer showing the highest swelling value.

Furthermore, temperature could also influence the swelling capacity. For coating materials A and C, their swelling degree decreased with the rise in temperature before the addition of the cross-linking agent, while those with added cross-linker did not display similar regularity. Notably, coating material B with the cross-linker formed at 60 °C exhibited the minimal swelling capacity (2.54%).

The glass transition temperatures (*T_g_*) of the aforementioned coating materials showed a graded variation (Table 2). The *T_g_* values increased progressively from material A to C, both with and without the addition of the cross-linker. Specifically, the *T_g_* range was 10.23 °C to 25.81 °C before cross-linking and 10.95 °C to 27.68 °C after cross-linking. It can be observed that the cross-linker had minimal impact on the *T_g_* for most materials, with the exception of material C. This suggests that the cross-linker can be utilized to enhance the hydrophobicity of the coating materials without significantly altering their thermal transition properties.

### 3.2. Surface Morphology and Spectral Characterization of Coated Films

**Figure 2:**
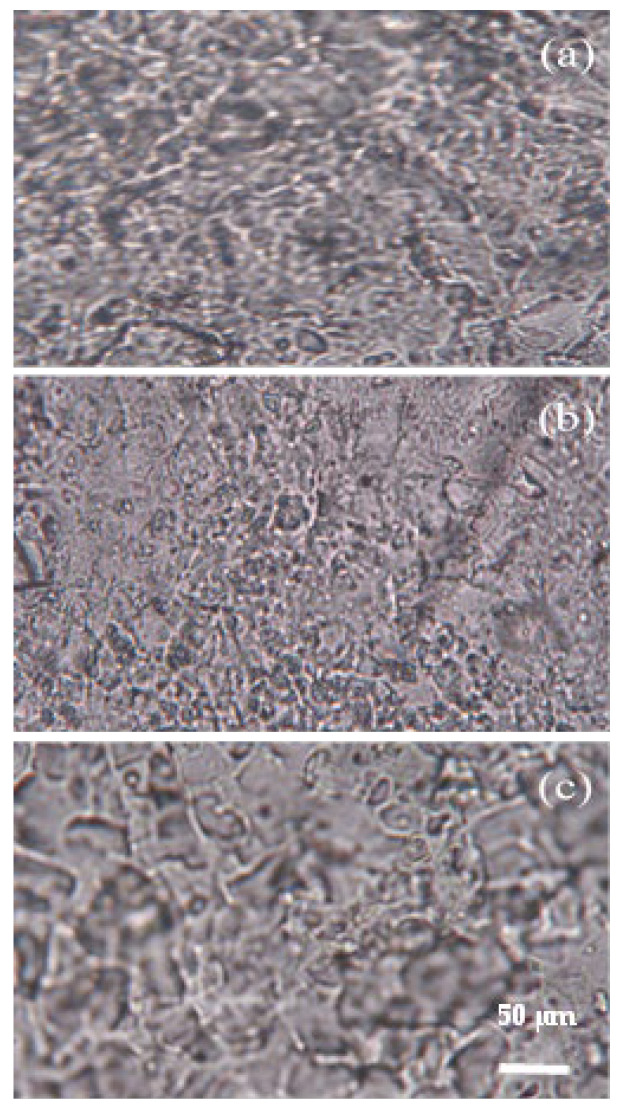
Micrographs of coatings removed from coated fertilizers (**a**): material A; (**b**): material B; (**c**): material C.

The surface morphology of the fertilizer coating prepared with the three modified materials is shown in Figure 2. By comparison, the coating surface of fertilizer-based material B was the most uniform and dense, with relatively few pores. The surface uniformity and porosity of fertilizer A were ranked at the medium level, while the smoothness of fertilizer C’s coating was remarkably inferior to the other two, with a higher proportion of large pores.

**Figure 3:**
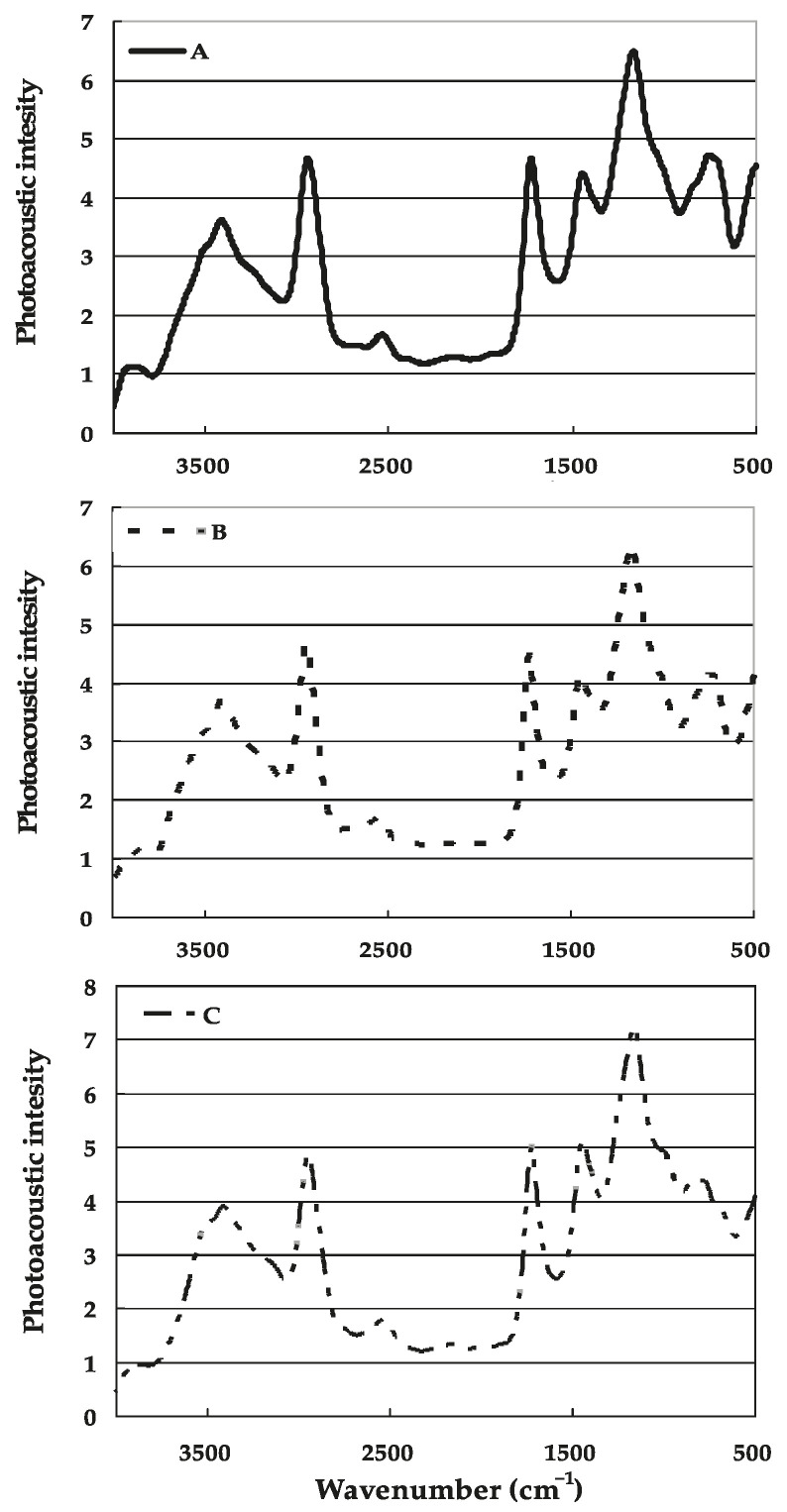
Fourier transform infrared photoacoustic spectra of coated films. A, B and C denote the fertilizer coating made from latex A, B and C with 2 wt% cross-linker, respectively, in the wave number range of 500~4000 cm^−1^ at moving-mirror velocities of 0.31 cm·s^−1^.

The three kinds of fertilizer coatings displayed quite similar FTIR-PAS spectra with abundant absorption in the range of 500~4000 cm^−1^ (Figure 3). The main absorption bands were observed in three regions: 2800~3450 cm^−1^, 1400~1750 cm^−1^, and 900~1200 cm^−1^. Based on the analysis of the monomers used in the polymer membrane synthesis, the relatively broad characteristic peak in the 3250~3550 cm^−1^ region is attributed to O–H and N–H stretching vibrations, and another characteristic peak appearing around 2930 cm^−1^ was generally assigned to aliphatic C-H stretching vibration, including both asymmetric and symmetric vibration. The absorption band at 1688 cm^−1^ proved the presence of C=O (carbonyl group). Additionally, the strong characteristic peaks at 1209 cm^−1^ and 1032 cm^−1^ represented C-O and Si-O vibration, respectively [36].

### 3.3. Nutrient Release in Water

**Figure 4:**
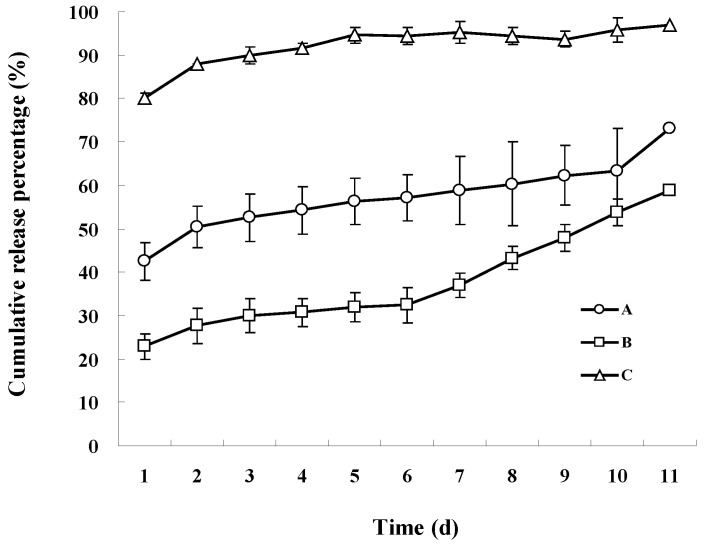
The effect of coating material (A, B and C with 2 wt.% crosslinker under the same coating ratio) on cumulative release of nutrients from the coated fertilizers in 40 °C static water. Error bars indicate the standard deviation of the means (*n* = 3).

The cumulative release of nutrients into static water from the encapsulated fertilizer (A, B, and C) is exhibited in Figure 4. Within one day, the cumulative nutrient release rates of the fertilizers coated with materials A and B were approximately 40% and 20%, respectively. In contrast, the cumulative nutrient release rate of the fertilizer coated with material C reached 80%. The results indicate that material C lacks controlled-release functionality. Furthermore, the data demonstrated that the release rate of fertilizer A was consistently faster than that of fertilizer B throughout the experiment.

Upon completion of the release experiments, fertilizer coated with material B released only about 60% of the total nutrients, whereas material A-coated fertilizer released over 70%. Conversely, fertilizer coated with material C released nearly 100% of the nutrients. These results indicate that CRF B exhibited the longest release duration, while CRF C demonstrated the fastest nutrient release among the three coatings.

### 3.4. Effects of Different Slow-/Controlled-Release Fertilizers on Rice Yield

The influence of different types of slow-/controlled-release fertilizers on the yield components of rice, including panicles per unit area, grains per panicle, seed-setting rate, and 1000-grain weight, is presented in Table 3. The average theoretical yields of F1, F2, F3, F4, F5, and F6 were 8.63 t·ha^−1^, 8.64 t·ha^−1^, 7.90 t·ha^−1^, 7.60 t·ha^−1^, 9.84 t·ha^−1^, and 7.72 t·ha^−1^, respectively, in 2020, which was generally higher than those in 2019. The theoretical yield of F5 ranked first for two consecutive years. Compared to conventional practices and sulfur-coated urea (Kingenta), the encapsulated fertilizer developed in this study enhanced all yield determinants to varying degrees, resulting in average theoretical yield increases of 27.0% and 29.4% in 2020, respectively. Furthermore, the CRF based on the material developed herein demonstrated excellent controlled-release performance, achieving the highest values in panicle number (322.5 thousand·ha^−1^), grains per panicle (126), and projected yield (9.84 t·ha^−1^) in 2020.

**Figure 5:**
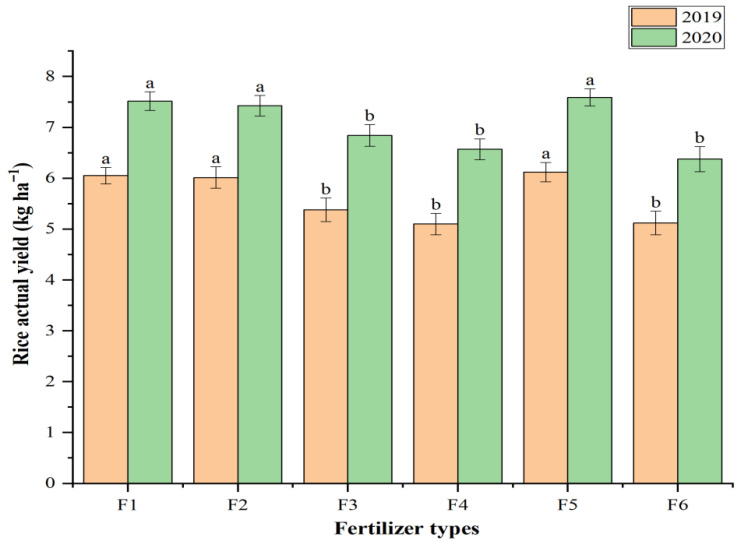
The impact of application of various slow/controlled release fertilizers on late rice actual yield in 2019 and 2020 in Yujiang. Different lowercase letters mean significant differences at 0.05 level for fertilization in the same year. Error bars indicate the standard deviation of the means (*n* = 3). Descriptions of the treatment names are given in Table 1.

The measured actual yield of late rice (Nongxiang 98) under the application of different types of slow-/controlled-release fertilizers, including the basal application of conventional compound fertilizer, is shown in Figure 5. The actual yield of rice under the application of the coated fertilizer developed in this study was higher than that of any other tested slow-/controlled-release fertilizer for two consecutive years. F5, especially, recorded a significantly (*p* < 0.05) higher value than F3 and F4, increasing by 13.8% and 20.0% in 2019 and 11.0% and 15.5% in 2020, respectively. Compared to the control (F6), the rice yield significantly (*p* < 0.05) increased by 19.5% in 2019 and 19.1% in 2020, demonstrating a marked effect on yield improvement.

## 4. Discussion

### 4.1. Impacts of Cross-Linker and Temperature on the Swelling Capacity

The swelling capacity of a coated film is a critical factor for its controlled-release applications. In our investigation, it was observed that the cross-linker could significantly (*p* < 0.05) decrease the swelling capacity of the three kinds of coating materials, especially at 60 °C, demonstrating that the cross-linking reaction could enhance the hydrophobicity of a material at the appropriate temperature. This might be ascribed to the fact that hydroxyl groups in polyacrylate were consumed by converting hydroxyl groups to esters during the cross-linking reaction. Consequently, the number of OH groups to establish hydrogen bonds with water molecules decreased in the cross-linked polyacrylate, which reduced the swelling degree of the membrane [34]. Furthermore, the selected 2 wt.% cross-linker ratio was based on a prior study [35].

**Figure 6:**
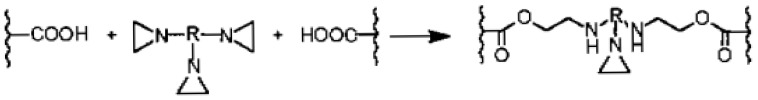
The sketch crosslinking reaction.

The chosen aziridine cross-linker contains multiple highly reactive rings. Its mechanism involves ring-opening and covalent bonding with the carboxyl functional groups of the polymer (Figure 6). Since a single cross-linker molecule can react with multiple polymer chains, it serves to create an extensive three-dimensional network. This structure is responsible for the enhanced flexibility and superior film-forming properties observed. Critically, the extent of cross-linking directly dictates the material’s final properties; a more complete reaction results in a denser, more defined network, thereby enhancing its hydrophobicity [37].

Variations in the membrane structure with film-forming temperature may account for the observed differences in swelling capacity. The distinct swelling behaviors of materials A and C before and after cross-linker addition are likely attributable to differing degrees of cross-linking achieved at their respective formation temperatures. It should be noted that material C yielded films with the lowest flexibility and relatively poor film-forming properties among the three materials, which can be explained by its higher *T_g_* (Table 2). This also led to the lowest hydrophobicity prior to cross-linker incorporation.

Overall, film materials used as CRF coatings should be of appropriate swelling capacity. A low degree of swelling may result in an unacceptably slow nutrient release rate and, in extreme cases, could completely inhibit release. Conversely, excessive swelling capacity may lead to an overly rapid release of nutrients, thereby undermining the controlled-release functionality [35]. Therefore, the release duration of CRF can be regulated by adjusting its swelling capacity, which should be maintained below 10% [38]. Based on the above results, it can be concluded that formulation B, containing the 2 wt% cross-linker and particularly formed at 60 °C, more effectively reduces nutrient release compared to the other formulations.

### 4.2. Impacts of T_g_ on the Coating

Generally, below the *T_g_*, the polymer is hard, rigid, brittle, and glass-like; above the *T_g_*, the polymer is soft, tough, rubbery, and flexible. Polymers whose *T_g_* is around 15 °C are usually considered to be appropriate coating materials [39]. An excessively low glass transition temperature (*T_g_*) promotes agglomeration among fertilizer granules, compromising both fluidization behavior and coating uniformity. In contrast, an overly high *T_g_* yields an excessively brittle membrane that inhibits effective cross-linking. Consequently, material B proved to be a superior candidate for coating fertilizers in a fluidized bed (Table 2). The tendency of the former coating material to become sticky can be attributed to its lower glass transition temperature (*T_g_*), ranging from 6.13 °C to 7.88 °C [35].

### 4.3. FTIR-PAS Analysis

There were abundant functional groups present on the coating surfaces, including C-O-C (1101 cm^−1^), C-O (1209 cm^−1^), C-H (2930 cm^−1^), C=O (1688 cm^−1^), and other functional groups in the FTIR-PAS spectra of the three coatings (Figure 3). These functional groups are critical for nutrient binding [36]. The main difference between them was the strength in the O-H vibration range (3250~3550 cm^−1^) and the remaining range (600~3200 cm^−1^), since their functional groups were similar (Figure 3). For the three kinds of fertilizer coatings, the ratio of absorption band denoting hydrophilic group O-H (3250~3550 cm^−1^) to that representing hydrophobic group aliphatic C-H (2800~2950 cm^−1^) took on the following order, B ≈ A > C, which means that the hydrophilicity intensity of the membrane was B ≈ A > C [39]. The results of the FTIR-PAS spectra can be used to compare the differences in hydrophobicity among these three materials due to different ratios of hard to soft monomers. Such a change slightly modified the molecular structure, especially for O-H. In light of the dynamic film formation process in a fluidized bed, a slight discrepancy was observed between the results for relative hydrophilicity from FTIR-PAS spectra and those for the swelling degree of the model membrane.

### 4.4. Nutrient Release of Polyacrylate-Coated Fertilizers

The results of the controlled-release study indicate that material B exhibited the best controlled-release performance, followed by material A, while material C demonstrated the poorest (Figure 4). Such distinct release patterns arise from differences in the hydrophobicity (Figure 1), surface morphology (Figure 2), and spectral characteristics (Figure 3) of the tested materials. What is more, different *T_g_* values can affect the coating quality to some extent.

The enhancement of hydrophobicity of fertilizer coating B with the cross-linker contributed to the slow release of nutrients (Figure 1), thanks to the three-dimensional structure in the cross-linking reaction. In addition, the observations of the surface morphology of the coated fertilizers prepared with the three modified materials, obtained through optical microscopy (Figure 2), can partially explain their nutrient release performance. Material B could form more coherent, homogeneous, and impermeable surfaces, as it has been reported that such structures could control the nutrient diffusion better [34]. Meanwhile, no special functional groups formed in the three modified materials (Figure 3), which did not change the other properties of the coating. Combined with the results of glass transition temperature (Table 2), material C, with the highest *T_g_*, enhanced the coating hardness and reduced the intergranular adhesion of the fertilizer particles. However, this came at the cost of a significant decline in its controlled-release performance. Although the coating prepared from material A exhibited improved hardness, its controlled-release efficacy was inferior to that of material B. In this study, material B with an intermediate *T_g_* demonstrated the best controlled-release performance. Therefore, when controlled-release performance is comparable, using a coating material with an appropriate *T_g_* is more advantageous.

### 4.5. Rice Yield

The application of CRFs in rice production presents a considerable challenge to their controlled-release performance, given the specific environmental conditions of paddy fields. In comparative analyses with other commercially available fertilizers of this category in China, CRFs based on the material developed in this investigation exhibited superiority for two consecutive years, attaining the foremost position in panicle number, grains per panicle, and projected and actual yield (Table 3, Figure 5). The seed-setting rates were only marginally lower than those achieved using fertilizers from the Shenyang Institute of Applied Ecology in 2019 and 2020 (Table 3).

The extreme climate in 2019 was extremely unfavorable to late rice. High-temperature heat damage and severe drought were the dominant factors leading to reduced yields. The climate in 2020 had an adverse impact on late rice in the early stage, but a favorable impact in the middle and late stages (Table S1, Figure 5). Despite the significant differences in weather conditions in 2019 and 2020, fertilizer developed in this study consistently achieved the highest yield, demonstrating its stable effectiveness. The yield advantage of this product in the experiment may be largely related to the unique characteristics of the rice growth environment, confirming that this product can still exhibit excellent controlled-release performance under long-term flooded conditions.

The waterborne polyacrylate-coated fertilizer achieved a stable yield (above 8 t·ha^−1^) for three consecutive years (2018~2020) at Yixing Base for Agri-Environment Research, Changshu National Agro-Ecosystem Observation and Research Station, Chinese Academy of Sciences, located in the Taihu Lake region (31°16′ N, 119°54′ E), China. The 3-year average yield (8.5 t·ha^−1^) of rice (Nanjing 8) was also significantly (*p* < 0.05) higher than that (7.1 t·ha^−1^) of a single application of urea with 240 kg N ha^−1^ [40]. These outcomes substantiate the considerable superiority and potential application prospects of the presently developed coated fertilizer within the context of practical rice cultivation. However, the efficacy of the CRF developed in this study needs to be supported by more robust evidence and validated through more extensive and long-term field trials.

## 5. Conclusions

The modified waterborne polyacrylate coating materials, especially material B, demonstrated effective control over nutrient release from soluble compound fertilizers and significantly minimized inter-particle adhesion. The hard-to-soft monomer ratio in the synthesis formulation was identified as a critical factor governing the structural and functional characteristics of the fertilizer coating. Among the coating latex formulations evaluated, only the one with an optimized hard-to-soft monomer ratio reached an appropriate glass transition temperature (*T_g_*) and displayed satisfactory controlled-release properties. These findings demonstrate that optimizing the composition of waterborne polyacrylate latex improves its performance as a coating material for fertilizers, making it particularly suitable for use in rice production systems.

## Figures and Tables

**Table 1:** The codes and types of different slow/controlled release fertilizers applied in field rice.

Code	Fertilizer Type	Nutrient Content
F1	Anhui Plasma Research Institute’s nano-fertilizer	N19-P11-K18
F2	Shenyang Ecology Institute’s rice-specific fertilizer	N26-P11-K11
F3	Institute of Soil and Fertilizer, Guangdong Academy of Agricultural Sciences: slow/controlled-release BB fertilizer	N23-P7-K20
F4	Sulfur-coated urea from Kingenta.	N43
F5	Nanjing Institute of Soil Science’s controlled-release fertilizer	N16-P10-K13
F6	Conventional compound fertilizer	N46-P12-K60

**Table 2:** The glass transition temperature (*T_g_*) and thermal capacity of model membranes based on materials A, B and C formed at 60 °C for 24 h.

Materials	A	B	C	A+	B+	C+
Sample weight (mg)	7.96	6.42	6.06	6.41	6.40	5.52
*T_g_* (°C)	10.23	14.87	25.81	10.95	15.34	27.86
Delta Cp (J/g . °C)	0.26	0.24	0.29	0.23	0.24	0.30

Note: + means the addition of 2 wt% cross-linker.

**Table 3:** The comparison of yield components of rice applied various slow/controlled release fertilizers in 2019 and 2020 in Yujiang.

Year	Code	Panicle Number (thousand·ha^−1^)	Grain Number per Panicle	Seed Setting Rate (%)	TGW (g)	Theoretical Yield (t·ha^−1^)
2019	F1	3021 ± 108 a	110 ± 13 ab	81.1 ± 2.7 a	27.65 ± 0.34 a	7.45 ± 0.21 ab
	F2	2985 ± 101 a	101 ± 15 b	84.9 ± 2.9 a	27.98 ± 0.46 a	7.16 ± 0.25 b
	F3	2870 ± 168 a	105 ± 16 b	80.4 ± 4.8 a	26.69 ± 0.71 b	6.47 ± 0.15 c
	F4	2889 ± 160 a	103 ± 19 b	74.8 ± 4.3 b	26.52 ± 0.62 b	5.90 ± 0.21 c
	F5	3025 ± 115 a	119 ± 11 a	81.9 ± 2.2 a	26.74 ± 0.41 b	7.88 ± 0.11 a
	F6	2892 ± 170 a	105 ± 18 b	75.1 ± 5.4 b	26.53 ± 0.40 b	6.05 ± 0.23 c
2020	F1	3135 ± 132 a	114 ± 11 ab	82.9 ± 3.1 a	29.32 ± 0.34 a	8.63 ± 0.13 ab
	F2	3105 ± 126 a	110 ± 12 b	86.5 ± 2.9 a	29.28 ± 0.46 a	8.64 ± 0.18 ab
	F3	2790 ± 130 a	119 ± 14 ab	81.2 ± 3.6 a	29.39 ± 0.51 a	7.90 ± 0.15 b
	F4	2895 ± 146 a	123 ± 16 a	75.3 ± 4.3 b	28.35 ± 0.42 b	7.60 ± 0.21 b
	F5	3225 ± 112 a	126 ± 10 a	83.8 ± 2.2 a	28.94 ± 0.32 ab	9.84 ± 0.11 a
	F6	3000 ± 151 a	117 ± 16 ab	76.6 ± 5.4 b	28.65 ± 0.56 ab	7.72 ± 0.23 b

Note: Different lowercase letters mean significant differences at 0.05 level for fertilization in the same year. Error bars indicate the standard deviation of the means (*n* = 3). Descriptions of the treatment names are given in Table 1.

## Data Availability

The original contributions presented in this study are included in the article/Supplementary Materials. Further inquiries can be directed to the corresponding authors.

## Supplementary Materials

The following supporting information can be downloaded at: https://www.mdpi.com/article/10.3390/polym17212816/s1, Figure S1: The production process of polyacrylate coating emulsion; Table S1: The formulation of waterborne polyacrylate coating; Table S2: The monthly precipitation and monthly average temperature during the growing period of late rice in 2019-2020.

## Abbreviations

The following abbreviations are used in this manuscript:

CRF	Controlled release fertilizer
SRF	Slow release fertilizer
NPK	Nitrogen, phosphorus and potassium
SAPs	superabsorbent polymers
PAs	Poly(acrylates)
N	nitrogen
TGW	Thousand-grain weight
