# Supplementary material for: Optimizing Waterborne Polyacrylate Coating for Controlled-Release Fertilizer Using a Wurster Fluidized Bed and Its Effects on Rice Yield
Source: Polymers (Basel). 2025 Oct 22;17(21):2816. doi: 10.3390/polym17212816 (PMC12609992; doi:10.3390/polym17212816)
Supplement: Supplementary file 1 [file polymers-17-02816-s001.zip › polymers-3920230-supplementary.pdf]

Table S1. The formulation of waterborne polyacrylate coating.

Component Classification	Selected Reagents	Mass Ratio Range (%)
Coating Solution Monomer	Methyl Methacrylate<br>n-Butyl Acrylate	15 ~ 25
	Acrylic Acid	15 ~ 25
	Methacrylic Acid<br>Acrylamide	1 ~ 3
	Vinyltrimethoxysilane	1.0 ~ 1.5
Emulsifier	Sodium Vinyl Sulfonate	0.5 ~ 0.7
	Polymeric Surfactant DNS-86	0.1 ~ 0.3
Crosslinking Agent	Aziridine and nano-zinc oxide	1 ~ 3
Catalyst	Ammonium Persulfate or Potassium Persulfate	0.3 ~ 0.5
Buffer	Sodium Bicarbonate	0.03
Antifreeze	Propylene Glycol	0.5
pH Regulator	Aqueous Ammonia	appropriate amount

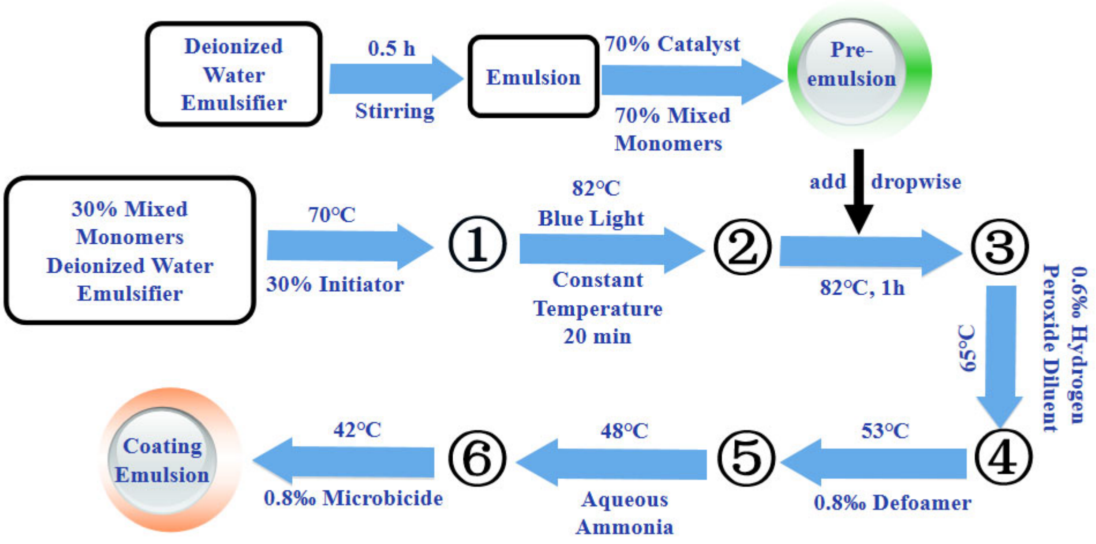

①—⑥intermediate products of the reactor reaction

Figure S1. The production process of polyacrylate coating emulsion.

Table S2. The monthly precipitation and monthly average temperature during the growing period of late rice in 2019-2020.

Item<br>Month	2019		2020	
	Precipitation (mm)	Average Temperature (℃)	Precipitation (mm)	Average Temperature (℃)
April	280	25.8	350	26.0
May	100	29.5	120	29.8
June	85	29.2	95	29.5
July	65	25.8	70	25.5
August	50	20.5	50	20.2
September	70	15.2	60	15.0
Period Average	108.3	24.3	124.2	29.5
